# Deintensification of Adjuvant Treatment After Transoral Surgery in Patients With Human Papillomavirus-Positive Oropharyngeal Cancer: The Conception of the PATHOS Study and Its Development

**DOI:** 10.3389/fonc.2019.00936

**Published:** 2019-10-01

**Authors:** Sarah Hargreaves, Matthew Beasley, Chris Hurt, Terry M. Jones, Mererid Evans

**Affiliations:** ^1^Velindre NHS Trust, Cardiff, United Kingdom; ^2^University Hospitals Bristol NHS Foundation Trust, Bristol, United Kingdom; ^3^Centre for Trials Research, Cardiff University, Cardiff, United Kingdom; ^4^Department of Molecular and Clinical Cancer Medicine, University of Liverpool, Liverpool, United Kingdom

**Keywords:** HPV-related head and neck squamous cell carcinoma, PATHOS, deintensification, transoral surgery, adjuvant

## Abstract

PATHOS is a phase II/III randomized controlled trial (RCT) of risk-stratified, reduced intensity adjuvant treatment in patients undergoing transoral surgery (TOS) for human papillomavirus (HPV)-positive oropharyngeal squamous cell carcinoma (OPSCC). The study opened in the UK in October 2015 and, after successful recruitment into the phase II, transitioned into phase III in the autumn of 2018. PATHOS aims to establish whether the de-intensification of adjuvant treatment in patients with favorable prognosis HPV-positive OPSCC will confer improved swallowing outcomes, whilst maintaining high rates of cure. In this article, we will outline the rationale for the study and how it aims to answer fundamentally important questions about the safety, effectiveness and functional outcomes of minimally invasive TOS techniques followed by adjuvant radiotherapy (RT) or chemo-radiotherapy (CRT) in this patient population.

## Introduction

PATHOS is a phase II/III randomized controlled trial (RCT) of risk-stratified, reduced intensity adjuvant treatment in patients undergoing transoral surgery (TOS) for human papillomavirus (HPV)-positive oropharyngeal squamous cell carcinoma (OPSCC). The study opened in the UK in October 2015 and, after successful recruitment into the phase II with a primary objective of swallowing function at 12 months (MD Anderson Dysphagia Inventory, MDADI), transitioned into phase III in the autumn of 2018 with co-primary endpoints of overall survival and MDADI at 12 months. PATHOS aims to establish whether the de-intensification of adjuvant treatment in patients with favorable prognosis HPV-positive OPSCC will confer improved swallowing outcomes, whilst maintaining high rates of cure. In this article, we will outline the rationale for the study and how it aims to answer fundamentally important questions about the safety, effectiveness and functional outcomes of minimally invasive TOS techniques followed by adjuvant radiotherapy (RT) or chemo-radiotherapy (CRT) in this patient population.

## Explanation of Trial Rationale

### Improved Prognosis of HPV-Positive Oropharyngeal Carcinoma

OPSCC is increasing in incidence worldwide due to infection with HPV, predominantly genotype 16. Mehanna et al. reported that 70% of OPSCC currently diagnosed in Europe are HPV-positive (1) although areas of Europe outside of the UK and Northern Europe were under-represented in this study and there is a lack of data for other countries, e.g., in Eastern Europe.

Patients with HPV-positive OPSCC have a significantly better prognosis, with a 58% reduction in the risk of death compared to patients with HPV-negative OPSCC (2). Patients tend to be younger and fitter and have 3 year overall survival (OS) rates in the order of 90%, although rates vary with stage and are influenced by other factors, including smoking history (2–4). This improved prognosis is reflected in the new TNM staging system, TNM8, which separates HPV-positive OPSCC from HPV-negative OPSCC in its prognostic staging groups (5). HPV-positive OPSCC appears to be more radiosensitive than HPV-negative OPSCC and HPV positive cell lines have increased radiosensitivity *in vitro* as a result of impaired DNA repair mechanisms (6, 7). Patients with HPV-positive disease have a higher response rate to radiotherapy alone (8), (induction) chemotherapy (9), and chemo-radiotherapy (9), compared to patients with HPV-negative disease.

Current treatment protocols for the management of OPSCC do not take HPV status into account and standard treatments, outside of clinical trials, are similar irrespective of HPV status. This is wholly appropriate until clinical trials prove that treatment may be modified in patients with HPV-positive disease. The importance of not modifying treatment prematurely is demonstrated by the recently published RTOG 1016 (10) and De-Escalate HPV (11) studies both of which reported superior outcomes with standard of care Cisplatin-based CRT compared to Cetuximab and radiotherapy in patients with HPV-positive OPSCC. Definitive results from other de-intensification/protocol modification trials in patients undergoing primary non-surgical management for HPV-positive oropharyngeal cancer are awaited. The importance of waiting for such data is reflected in the recommendations of The National Institute for Clinical Excellence (NICE). NICE is a UK based organization which provides advice and guidance concerning the improvement of health and social care. NICE guidelines on management of patients with cancers of the upper aerodigestive tract^1^, which were updated in 2016, have a section on De-intensification of treatment in HPV-positive OPSCC which states: “Do not offer de-intensification of curative treatment to people with HPV-positive cancer of the oropharynx, unless it is part of a clinical trial.” Carefully planned and well-monitored studies are therefore a fundamental part of the effort to improve future treatments for HPV-positive OPSCC. This is reinforced by the recently published ASCO provisional clinical opinion by Adelstein et al. (12) which concludes that whilst the prognostic ability of the 8th edition of the American Joint Committee on Cancer Staging System is considered to be strong and reflective of the current outcomes of treatment for HPV-positive OPSCC “careful study and the analysis of well-designed clinical trials” are mandatory prior to altering current standard practice in this group of patients.

### Transoral Surgery for Oropharyngeal Cancer: A New Treatment Paradigm

Transoral Surgery (TOS), including non-endoscopic methods such as the Huet procedure ie, TOS with a monopolar cautery, as well as endoscopic-assisted transoral surgery—Transoral Laser Microsurgery (TLM) or Transoral Robotic Surgery (TORS)—are minimally invasive surgical procedures, usually performed with a neck dissection, which have become more widely adopted for the treatment of OPSCC over the last 10–15 years (13–15). These techniques have the potential to excise OPSCCs with significantly less functional morbidity (16) compared to traditional, open access surgical techniques (17). Two systematic reviews and a meta-analysis (18–20) have reported similar oncological outcomes from TOS compared to primary RT/CRT, albeit with a different profile of adverse events.

The ORATOR trial (NCT01590355) (21) presented earlier this year is the first study we are aware of which randomized patients with OPSCC to either trans-oral robotic surgery or primary radiotherapy ± concurrent chemotherapy. Between 2012 and 2017, 68 patients were randomized, 34 in each arm. P16 status was positive in 88% of patients. The results have only been published in abstract form so far after a median follow-up of 27 months. MDADI scores were statistically superior in the radiotherapy group (*P* = 0.042), although did not meet the definition of a Clinically Meaningful Difference (CMD). For all other QOL of life metrics, outcomes were similar at 12 months and overall and progression-free survival outcomes were also similar. We await the comprehensive results of this small study with interest, as well as data on the proportion of patients who received adjuvant treatment following TORS. It is important to highlight that the question of de-escalation of adjuvant therapy was not addressed in this study (unlike PATHOS).

### Adjuvant (Post-Operative) Treatment for OPSCC

Surgery for OPSCC is followed, in most cases, by adjuvant treatment with either post-operative radiotherapy (PORT) or post-operative chemo-radiotherapy (POCRT). In reality, the optimal adjuvant treatment following TOS is not well-defined, particularly in the context of HPV-positive OPSCC.

Standard post-operative radiotherapy (PORT) protocols are based on the results of studies such as RTOG 73-03 (22) (reported over 30 years ago) that demonstrated improved local control in locally advanced head and neck cancers following PORT (23). A subsequent randomized study (24) explored the optimum dose for PORT and recommended a minimum dose of 57.6Gy to the operative primary tumor bed and involved lymph nodes areas, with a dose of 63Gy in 1.8Gy fractions limited to sites at higher risk, particularly areas of extracapsular spread (ECS). Updated long term data from this study did not, however, show a dose response relationship above doses of 57Gy in patients with good prognosis disease (based on a cumulative points score) whereas poor prognosis patients required 60–63Gy (25). Not surprisingly there is significant variation in practice; in the UK, most centers recommend a dose of 60Gy in 30 fractions over 6 weeks for the majority of patients, with an optional boost to high risk areas (ECS and/or positive margins) up to 66Gy (26). It is important to appreciate that the historical studies, upon which the doses of PORT we prescribe today are based, would have had a relatively low proportion of patients with HPV-positive OPSCC, because of the lower prevalence of HPV in OPSCC at the time and also the inclusion in these studies of multiple head and neck cancer subtypes, not only OPSCC. This observation is also true when considering the use of POCRT. Current practice has been informed by the results of two international studies (EORTC 22931/RTOG 9501). Pooled results from these studies (27) demonstrated improved overall survival (OS) in patients with positive (or “involved”) surgical resection margins around the primary tumor and/or presence of ECS in nodal disease of the neck and these pathological features are now widely used criteria for POCRT. These studies included patients with squamous cell cancers from a variety of head and neck sub-sites recruited between 1994 and 2000 and, based on HPV prevalence rates at that time (1), we estimate that <100 patients (~13% of study cohorts) recruited to these studies would have had HPV-positive OPSCC. Consequently, it is unclear how relevant these results are to patients with HPV-positive OPSCC.

There is a large body of evidence demonstrating an excess of acute and late toxicity attributable to adjuvant treatment following TOS. In a prospective cohort study of 111 patients (28), the 13 who had TORS alone did not require a gastrostomy tube and had significantly higher eating scores at 3 and 6 months compared to those who received adjuvant treatment. The 60% (67/111) who received POCRT had consistently worse overall QOL scores until 6 months after completion of treatment. Gastrostomy tube use was doubled by adjuvant treatment in the largest TLM series (13), including 204 patients, from 17 to 33%. Nineteen percent were still dependent upon a gastrostomy tube a year after treatment. In 66 OPSCC patients treated with TORS (32), 97% were managing an oral diet a month after surgery, but subsequently 27% (18/66) required a gastrostomy tube during their adjuvant therapy and three (4.5%) remained dependent upon a tube for more than 2 years after treatment. A similar outcome was observed in 81 patients treated with TORS (33). All patients were discharged postoperatively on a full oral diet, but 13 (16%) required gastrostomy tube placement during adjuvant treatment. Five patients were still dependent upon a tube over a year later. Factors which predict for tube dependency include age (>55 years) and the extent of TORS resection. A high T stage (pT3/pT4) predicted the need for permanent dependency upon tube feeding.

The addition of chemotherapy to adjuvant radiotherapy predicts for worse functional outcomes compared to adjuvant radiotherapy alone. In 38 OPSCC patients, speech, diet and eating scores at 6 and 12 months after treatment were significantly higher following TORS without adjuvant treatment vs. TORS followed by PORT. Scores following POCRT were worse again (34). In addition to the above, a systematic review of TORS for OPSCC demonstrated a significant deterioration for a range of swallowing outcome measures in those who had chemotherapy in addition to adjuvant radiotherapy (35).

### Rationale for De-Intensification

It is known that toxicities are strongly linked to the dose of radiotherapy (RT) received by the normal tissues. Severe late toxicities have been reported in 43% of patients after treatment with primary CRT, albeit in the pre-IMRT era, and toxicity of this magnitude can last a lifetime (36). The key late toxicity which has the greatest impact on QoL is dysphagia (difficulty swallowing) (37). Dysphagia is an independent predictor of poor QoL and correlates with the mean dose of RT received by swallow-related organs, especially the superior pharyngeal constrictor muscles (PCM) and the supraglottic larynx (38). Chronic dysphagia is a complex problem; one study looked at a consecutive series of patients who had all been referred for modified barium swallow for the investigation of chronic dysphagia up to 5 years following definitive RT/CCRT for head and neck cancer. The majority of cases were oropharyngeal. Eighty six percent (25/29) of these patients suffered repeated episodes of aspiration pneumonia with half of these cases warranting hospitalization (39).

In view of the fact that HPV positive OPSCC patients are younger, and more likely to survive for many years following their diagnosis and treatment, efforts to explore the potential of de-intensified treatment strategies in order to reduce long-term toxicities are at the forefront of current head and neck oncology research (see Table 1 for a summary). Despite the negative results from two phase III studies which have substituted Cisplatin for Cetuximab (10, 11), early results from phase II studies evaluating other strategies for toxicity reduction have reported promising results.

**Table 1:** Summary of completed relevant trials mentioned in this review.

**Trial reference**	**Type of trial**	**Patient numbers**	**Topic**	**Conclusion**
RTOG 1016 (10)	Randomized Phase 3	849	Cisplatin/RT vs. Cetuximab/RT	Superior outcomes in Cisplatin/RT group
DeEscalate HPV (11)	Randomized Phase 3	334	Cisplatin/RT vs. Cetux/RT	Superior outcomes in Cisplatin/RT group
ORATOR (21)	Randomized Phase 3	68	TORS vs. primary RT ± adjuvant treatment	At 27 mths follow up superior outcomes in primary RT group
Sethia et al. (28)	Prospective cohort	111	TORS ± POCRT	POCRT reduces QoL up to 6 months post-treatment
ECOG 1308 (29)	Single arm phase 2	80	3 × IDC with cCR 54Gy/27F + Cetux	Good outcomes in favorable risk groups
Chen et al. (30)	Single arm phase 2	44	2 × IDC with cCR or cPR 54Gy/27F + weekly pac	Good outcomes and reduced toxicity
Chera et al. (31)	Single arm phase 2	44	60Gy/30F with weekly 30 mg/m^2^ cis	Comparable oncological control and reduced toxicity

Two trials have explored the potential for reduced dose radiotherapy following response to induction chemotherapy. ECOG 1308 (29) was a single arm phase II study which showed promising outcomes with reduced dose radiotherapy (54Gy in 27 fractions and Cetuximab) in 80 patients with a complete response to 3 cycles of induction chemotherapy (TPF: Docetaxel, Cisplatin and 5-FU). Although 2 year Progression Free Survival (PFS) of 80% (95% CI: 65-89) was lower than expected, *post-hoc* analysis suggested extremely good outcomes (2 year PFS and OS of 96% [95% CI: 76-99]) in favorable-risk patients (TNM7: T1-3 N1-N2b, <10 pack/year smoking history). Another single arm phase II study of 44 patients with stage I-III (TNM8) HPV-positive OPSCC (30) gave 54Gy in 27 fractions of radiotherapy to the primary tumor and involved nodes and 43Gy to the prophylactically treated uninvolved nodes, with weekly Paclitaxel chemotherapy in patients who had a complete or partial response to 2 cycles of induction chemotherapy (Carboplatin and Paclitaxel). The trial demonstrated a PFS rate of 92% (95% CI 77–97%). This compared favorably with historical studies, in addition to a reduced toxicity profile. Whilst the results of small, non-randomized studies must be interpreted with caution, they suggest that the strategy of reduced dose radiotherapy after induction chemotherapy warrants further evaluation in larger studies.

Other studies have investigated the role of reduced dose radiotherapy in HPV-positive OPSCC, without prior use of induction therapy. In one prospective single-arm phase II study 44 patients with stage I-II (TNM7) HPV-positive OPSCC and a minimal smoking history were treated with reduced dose IMRT and reduced dose concomitant weekly cisplatin (31, 40). In total 43 patients underwent the surgery and were included in the analysis. Biopsies of tumor sites following treatment demonstrated complete pathological response rate of 98% (40/41) at the primary site (2 patients were T0) and 84% (33/39) in the neck nodes. The biopsy-positive primary site was resected and no viable tumor found. Treatment was associated comparable oncological control and reduced toxicity rates when compared with contemporary studies (e.g., PARADIGM) where patients received 70Gy of radiation.

The NRG HN-002 study uses a lower dose of radiotherapy to treat good prognosis HPV-positive OPSCC. Patients were randomized to receive reduced dose IMRT, 60Gy in 30 fractions over 6 weeks with concurrent weekly Cisplatin (40 mg/m^2^) or moderately accelerated reduced dose IMRT alone, 60Gy in 30 fractions over 5 weeks. The study is currently in follow-up.

The possibility that TOS followed by reduced intensity PORT or POCRT may be used to reduce long term functional morbidity in patients with favorable prognosis HPV-positive OPSCC is currently being tested in the PATHOS study (ClinicalTrials.gov NCT02215265), a UK and European prospective, phase III, randomized controlled trial (RCT) (41).

## Trial Design and Justification

**Figure 1:**
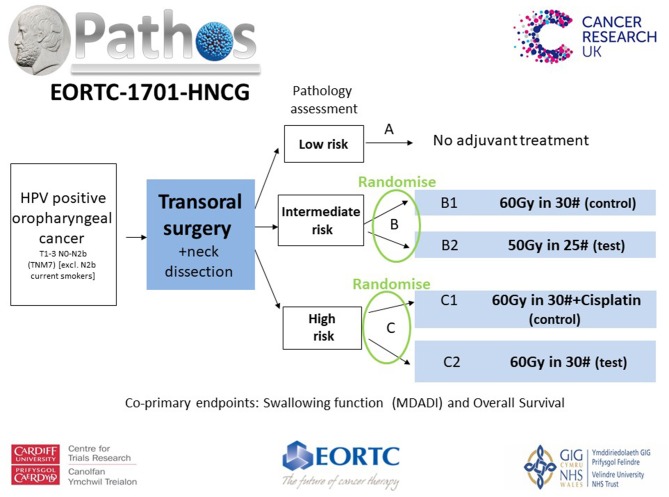
Patients with T1-3 N0-N2b (TNM7) HPV positive OPSCC undergo TOS and neck dissection before being stratified into risk groups based upon their pathology. Randomization takes place within the intermediate (Group B) and high risk (Group C) groups. Patients in Group B [T3/N2, PNI, vascular invasion (VI)] are randomized between the control arm of 60Gy in 30 fractions over 6 weeks of PORT and the test arm of 50Gy in 25 fractions over 5 weeks. Patients in Group C (involved <1 mm margins and/or ECS) are randomized between a control arm of POCRT, 60Gy in 30 fractions over 6 weeks with concurrent Cisplatin and the test arm of PORT alone, 60Gy in 30 fractions over 6 weeks. There is an option to boost high-risk sub-volumes to 66Gy in 30# over 6 weeks in Group C patients. The co-primary end points are patient-reported swallowing function (MD Anderson Dysphagia Inventory score) 12 months after treatment and overall survival. Secondary outputs include loco-regional control and quality of life.

The PATHOS (Postoperative Adjuvant Treatment for Human Papillomavirus (HPV)-positive Tumors) study, aims to demonstrate that risk-stratified de-intensification of adjuvant treatment maintains high survival and low recurrence rates in patients with HPV-positive OPSCC, but improves long term swallowing function (Figure 1).

Multiple studies have shown 50–60Gy to be a critical dose range concerning the risk of long term dysphagia when delivered to the pharyngeal musculature (42–44). A systemic review in 2017 (45) found that prognostic factors consistently associated with a risk of feeding tube dependency both short term and over 6 months after treatment included the dose received to the larynx, superior and inferior PCMs and the contralateral parotid gland. Threshold values for tube dependency beyond 6 months were a mean dose (Dmean) of 50.7Gy to the larynx and inferior PCMs. This is supported by normal tissue complication probability (NTCP) models for swallowing endpoints including a steep rise in the NTCP curve when the mean dose to PCMs reaches 50–55Gy (46). No threshold value was found for the contralateral parotid gland. In an overview of studies characterizing the effect of radiation on normal tissues in relation to the quality of life of head and neck cancer patients (47), dose thresholds to normal tissue structures were recommended on the basis of numerous studies which looked at both the physiology of xerostomia and dysphagia—the two symptoms which have the most negative impact on QoL of these patients. These included keeping a Dmean below 26Gy to the parotid gland and 39Gy to the contralateral submandibular gland. Generally a mean dose threshold of 50–60Gy was reported for the multiple pairs of muscles involved in swallowing. A dose less than this resulted in only very minor risk of aspiration. A recommendation was therefore made to keep the mean dose to the PCMs below 50Gy and <20Gy to the larynx. The Eisbruch Group (42) supported this with data showing when superior PCMs receive 56Gy there is a 25% risk of aspiration detected by VF, when they receive 63Gy this increases to 50%. Reducing post-operative radiotherapy (PORT) from 60 to 50Gy may therefore significantly reduce long term swallowing complications following treatment. The reduced dose of 50Gy being tested in Group B patients was recommended by the National Cancer Institute (NCI) Head and Neck cancer steering committee's clinical trials planning meeting in the postoperative setting following TORS of pharyngeal cancers (48). The same dose has been tested in the ECOG 3311 study (NCT01898494), a phase II US RCT which has also evaluated the role of reduced dose PORT in patients with “intermediate risk” pathological risk factors after TOS, with progression free survival (PFS) as its primary endpoint. ECOG 3311 has completed recruitment and is currently in follow-up.

In addition to PORT, adjuvant chemotherapy (POCRT) following TOS for OPSCC is also a significant factor influencing swallowing outcomes (13). Historically, POCRT has been advised for patients with tumors demonstrating high risk features including involved margins and/or ECS. This advice is based upon the combined results of studies such as EORTC 22931 and RTOG 9501 (27) which, as mentioned earlier, are not wholly representative of the HPV-positive demographic. Indeed, when there is a lack of soft tissue deposits, there is a lack of clarity in the literature to date as to whether ECS is prognostic in the context of HPV-positive OPSCC. Ultimately, there is a limited amount of prospective data on the prognostic nature of ECS and whether or not chemotherapy in addition to radiotherapy impacts upon outcomes. Some retrospective studies show ECS to be a poor predictor of recurrence (49, 50) whilst other retrospective studies support an association between ECS and inferior survival (51, 52). Furthermore, retrospective studies have not demonstrated any overall survival benefit with POCRT compared to PORT alone following TLM in patients with high-risk pathological features (53). In view of the significant increase in toxicity due to the addition of chemotherapy (including a 2% increase in treatment-related mortality), it is of upmost importance we are able to justify the inclusion of chemotherapy in adjuvant treatment regimes in the context of HPV-positive OPSCC. By randomizing patients with ECS to PORT vs. POCRT, PATHOS will provide much needed, important prospective data on this issue.

With the inclusion of TORS and TLM, PATHOS will add to another area where evidence for adjuvant treatment and functional outcomes is lacking. Open surgery plus adjuvant RT are associated with worse functional outcomes compared to primary chemo/RT but swallowing outcomes following transoral techniques for OPSCC are not well-reported. A systemic review and meta-analysis of over 500 patients in 17 retrospective studies treated with TORS concluded that treatment with TORS had a good rate of oncological control as well as a positive impact upon QoL and long term swallowing function (54). Two published retrospective series of patients treated with TLM and any appropriate adjuvant treatment, have demonstrated oncological control equivalent to non-surgical management in addition to good swallowing function outcomes when compared to non-surgical series. Rich et al. (53) published a series of 84 patients with stage III or IV OPSCC treated primarily with TLM. Eighty two percent were HPV positive and 92% received adjuvant treatment. At a minimal follow up of 2 years, disease specific survival at 2 years was 96% and at last follow up, 81% had acceptable swallowing function (normal–mild dysphagia). 3.4% remained dependent upon a gastrostomy tube for feeding. The second series (14) was larger and reported on 153 patients, of which 66% were HPV positive. OS at 3 years was 84.5% and tube dependency at 1 year was 1.3%. Both series concluded that TLM offered equivalent oncological control to non-surgical options in this setting, and demonstrated superior swallowing function outcomes when compared to published non-surgical data. PATHOS will allow us an opportunity to prospectively investigate whether we can build upon these positive outcomes by including a similar demographic of patient cohorts who are treated following TORS with de-escalated adjuvant therapy. To date, apart from the early data from ORATOR, there has not been any prospective randomized data on functional and cancer-specific outcomes in OPSCC treated with TLM or TORS.

## Trial Outcome Measures

In order to inform research priorities, two head and neck patient focus groups were consulted and strongly agreed that there was a lack of research concerning swallowing outcomes. They particularly highlighted the need for focusing on the prevention of dysphagia. A PATHOS patient engagement group has been formed, including 5 patients who have had transoral resection for OPSCC either with or without adjuvant treatment. They all agreed that the adjuvant treatment was much harder to get through compared to the initial transoral resection, and was the source of the majority of significant and long term side effects.

The primary outcome of the phase 2 and co-primary endpoint of the phase III is long term swallowing function, assessed by the MDADI at 12 months. This particular time point was chosen as the aim is to analyze long term swallowing outcomes. Longitudinal data (55) demonstrates that the majority of functional recovery is seen by 12 months with minimal amounts of recovery seen between 12 and 24 months. MDADI is psychometrically validated, sensitive to longitudinal changes and its trajectories follow expected patterns in distinguishing between different cancer treatment regimens (56, 57). It is widely used in clinical practice and has been included as an outcome measure in a range of UK and international trials such as ECOG 3311 (NCT01898494), NRG HN002 (NCT02254278), DARS (58), Best-of (NCT02984410), De-Escalate (11), and TORPEdO (NCT03561220). A 10 point difference between groups in the MDADI score differentiates aspirators from non-aspirators, tube-dependent from oral eaters and clinically distinct diet levels (59) and has been set as the Minimum Clinically Important Difference (MCID) for the study.

The best objective measure of swallowing function is videofluoroscopy (VF) as it offers a real time visualization of the oropharyngeal swallow, displaying the swallowing process from the lips to the upper esophageal tract. VF detects dysphagia endpoints which are clinically significant and may not otherwise be picked up on patient reported outcomes, such as aspiration. The recovery of swallowing after head and neck cancer treatment is multifaceted and differs both between clinical tests and clinician and patient-reported outcomes. No single measure of swallow function can replace another and the pathway of each assessment can alter throughout survivorship. With this in mind, as well as performing VF, PATHOS introduces a novel panel of swallowing assessments which the trial hopes to validate. The panel was developed in collaboration with internationally recognized experts in swallowing and with a wide range of experience of multi-center studies. To permit comparisons with a related trial, DARS (58), the swallowing panel has been adopted by the two studies. The panel includes the Performance Status Scale–Head and Neck (PSS-HN) (60); a 3-item clinician assessed measure of functional performance based on patient interviews. It is quick and inexpensive and has been adopted by HANA, the UK Head and Neck Audit. The Water Swallow Test (WST) is included which is quick and inexpensive, offering an effective way to monitor overall swallowing performance. Finally, the panel also includes the EORTC QLQ C30 (61) and HN35 (62) questionnaires which incorporate psychological, social, occupational and physical well-being to form a multidimensional assessment of the overall health-related quality of life of a patient.

PATHOS was designed as a Phase II study that would roll seamlessly into a Phase III. The Phase II was originally designed to prove feasibility of recruitment and superiority of swallowing function with deintensification with the Phase III being designed to prove non-inferiority of overall survival. Initially the Phase II stage of the study was funded by Cancer Research UK. As planned, once we proved feasibility of recruitment we applied for funding for the Phase III to prevent a hiatus in recruitment whilst we waited for the Phase II swallowing data to mature. The funding committee approved the Phase III funding but specified that the swallowing outcome should be moved to a co-primary endpoint of the Phase III. Consequently the trial is now in Phase III with the following design. Assuming:

1. Recruitment from Phase II and III takes 6.5 years
2. Follow up continues for a further 4 years
3. Overall survival in the control arms is 92% at 3 years (13)
4. Common exponential drop-out rate of 0.02

it is calculated that 574 patients and 94 events will be required to demonstrate that the 3 year overall survival is not worse than 87% [a 5% non-inferiority margin from the 92% in the control arm (equivalent to a hazard ratio of 1.795)], with power 80% and one-sided alpha of 5% [calculations done using nQuery + nTerim version 3.0 (63)]. For the co-primary endpoint of swallowing function, with 574 patients randomized, to find a difference of 10 pts (58) in MDADI with SD = 15, alpha = 0.05 two sided, we will have 99.99% power. So even if MDADI is completely independent of OS, the overall power for the co-primary will be 80%. Recruitment will continue until 574 patients have been randomized into Group C. We predict that this will be when ~1,100 patients have been recruited prior to surgery. We predict that fewer patients will be recruited into Group B (*n* = 210) but we intend to combine data with the ECOG 3311 trial (*n* = 377, estimated to complete in 2023, randomizing patients with “intermediate risk” pathology to 60Gy in 30 fractions or 50Gy in 25 fractions of RT, primary endpoint PFS), which should be sufficient to analyse the non-inferiority of OS in Group B in a pooled analysis. In order to assess the validity of pooling the data from the two trials, the OS in the control groups from the two trials will be compared prior to analysis. We predict that recruitment will continue until late 2022 with follow up for 4 years after that.

## Additional Benefits

PATHOS is the first UK study of post-operative IMRT for head and neck cancer and so presents an excellent opportunity to develop radiotherapy treatment quality assurance (RTTQA) in this area. Following discussion with the NCRI RTQQA group, a comprehensive RT-QA protocol has been developed for use within PATHOS. An additional novel aspect of PATHOS will involve the production of an atlas to guide the outlining of swallowing structures. Dose/volume data for the swallowing structures will be collected and for the first time compared to toxicity outcomes prospectively in a randomized trial. There is an ever increasing uptake of transoral surgical practice in the UK but as yet there is no current standardized practice for the treatment of OPSCC. PATHOS is a unique opportunity to standardize trans-oral techniques and establish surgical quality assurance (QA). Surgical QA and quality control (QC) will therefore be defined within a surgical guidance document developed for the PATHOS protocol. Finally, pathological reporting of HPV-positive OPSCC has not been standardized in the UK and PATHOS represents an opportunity to develop this. In addition, a National Cancer Institute Head and Neck Cancer Steering Committee Clinical Trials Planning Meeting on TORS of pharyngeal surgery in 2011 (48) requested the traditional pathological risk factors be re-evaluated in the context of HPV-positive disease. The PATHOS team have developed a pathology guidance document that clearly defines clear and close margins, and the need for marginal biopsies as part of the TLM procedure.

## International Collaboration

PATHOS is a Cancer Research UK (CRUK) funded study which initially opened in the UK as a phase II study in October 2015 and has recruited well, with 259 patients entering the study by 01/2019. With further support from CRUK, the study transitioned seamlessly into the phase III in October 2018, aiming to open across international sites in Europe and further afield, including Australia and the US. In Europe, the study will be conducted in collaboration with the European Organization for the Research and Treatment of Cancer (EORTC) Head and Neck group, as well as other international partners in France and elsewhere. This collaborative grouping will work together to recruit the ~1,100 patients required by 2022. If this is achieved, PATHOS will be a potentially practice changing trial, which could establish TOS followed by reduced intensity RRT/CRT as a standard of care for future patients with transorally resectable HPV positive OPSCC.

## Conclusion

The highly attractive prospect that treatment protocols can be de-intensified to minimize long term toxicity in patients with favorable prognosis OPSCC must be fully evaluated in prospective, randomized clinical trials before being adopted into routine care. PATHOS is an international collaborative trial which will help define the role of TOS followed by reduced intensity adjuvant RT/CRT in future treatment paradigms for this disease. Whilst the setting up of a trial of this magnitude and breadth is not a trivial undertaking, it will only be through continuous establishment of randomized controlled trials such as this that fundamental clinical questions can be answered in a safe and scientific manner in order to ultimately improve patient care and the quality of life experienced after treatment.

## Author Contributions

SH was lead author with contributions from MB, CH, TJ, and ME. MB, TJ, and ME contributed to the editing process.

## Conflict of Interest

The authors declare that the research was conducted in the absence of any commercial or financial relationships that could be construed as a potential conflict of interest.

## Footnotes

^1^www.nice.org.uk/guidance/ng36/chapter/recommendations/hpvrelated-disease
